# Identification of Research Priorities during the COVID-19 Pandemic: Implications for Its Management

**DOI:** 10.3390/ijerph182413105

**Published:** 2021-12-12

**Authors:** Jianhong Luo, Minjuan Chai, Xuwei Pan

**Affiliations:** Department of Management Science and Engineering, Zhejiang Sci-Tech University, Hangzhou 310018, China; cmjchris@163.com (M.C.); panxw@zstu.edu.cn (X.P.)

**Keywords:** research priority, COVID-19, pandemic, public health emergency management, text analysis

## Abstract

Novel coronavirus disease 2019 (COVID-19) pandemic has had a great impact on global production and life in the past period. Countless researchers devoted themselves to rescuing patients and reducing its impact. Analyzing the literature published during the pandemic and identifying the research priorities is of great significance to quickly discover research gaps, rationally allocate scientific research resources, and promote the development of the global research platform. To understand the swing of research priorities during the pandemic, this paper proposed a research priorities identification framework for pandemic based on scientific literature text analysis. Moreover, a research priority metric model was proposed to measure the characteristics of research priorities, and the empirical analysis from COVID-19 scientific literature was conducted to identify the research priorities during the pandemic. As a result, the research priorities identified by the method proposed in this paper discovered the fine-grained dynamic characteristics along with the process in the pandemic outbreak, and based on this, the emergency scientific research response strategies were discussed to give implications for the public health emergency scientific research and management.

## 1. Introduction

The recent outbreak of the Novel coronavirus disease 2019 (COVID-19) pandemic is a large-scale public health emergency with a huge impact and a serious threat to human health. On 11 March 2020, the World Health Organization (WHO) said that the COVID-19 outbreak had reached the stage where it can be described as a pandemic. In the context of the COVID-19 pandemic, domestic and foreign related scientific research have made rapid progress, and the number and speed of published research literature has increased. The research content of COVID-19 pandemic involves epidemiology [1,2,3,4], detection and diagnosis [5,6], vaccine and treatment [7,8], as well as social and economic impact [9,10,11]. At present, the research on pandemics is developing rapidly, but the research gap still exists [12], such as under-utilized information [13], inconsistent goals and strategies between countries [14], and high levels of research duplication [15]. Scholars around the world are committed to developing clinical treatment measures to control the pandemic faster, reduce casualties, and minimize socio-economic impact.

When a pandemic occurs, rationally constructing scientific research partnerships, scientifically distributing scientific research resources, conducting scientific research in an orderly manner, and rapidly producing and applying scientific research results are the key and focus of pandemic prevention and control. As is known, most research priorities were formulated by expert groups, relevant staff, and stakeholders based on experience, which can give reference and instruction for a pandemic. A hybrid method combining online surveys and expert group discussions [16], extensive stakeholder participation [17], and holding global research forums [18] were all used to determine research priorities, rationally allocate resources, and build a robust global research response. However, Andreas et al. argue that a rapid assessment of a dynamic research field such as COVID-19 requires an approach that is more direct and has a wider scope than that of the current gold standard methods, such as scoping and systematic reviews [19]. Due to the complexity and variability of pandemic outbreaks, it is significant to systematically and dynamically identify research priorities in each key stage of the pandemic development process, especially in the early stages of a pandemic. Since the outbreak of the pandemic such as COVID-19, there has been a surge in published COVID-19 research literature in the early stage [20], which provided scientific materials for the identification of emergency research priorities and can be considered as the key proxy of reflecting the trend of research in both theory and practice [21]. With the more and more extensive application of text analysis based on machine learning, it is possible to quickly analyze a large number of literatures [22,23]. Among them, the topic model [24,25], combined with the advantages of human insight and machine large-scale analysis, can be an important tool for literature analysis. Moreover, the use of sound methods and scientific processes is critical to ensuring the identification of the research priorities [26]. Therefore, through systematic literature data mining, it should be more significant and objective to identify emergency research priorities from both micro and macro levels. 

The contributions of this paper are: (1) a research priorities identification framework for pandemic based on scientific literature text analysis was proposed, which identifies the research priorities in pandemic systematically and shows more objective than the previous related studies. (2) A research priority metric model was proposed. The model defines three metrics from the perspective of overall focus, time urgency, and research resource investment, which can reveal the dynamic evolution characteristics of research priority in each stage of pandemic. (3) An empirical study for research priorities identification from the COVID-19 literature was conducted to demonstrate the effectiveness of the proposed framework and metric model, and to gain insights from the pandemic to improve the capability of public health emergency scientific research and management.

## 2. Method

**Figure 1:**
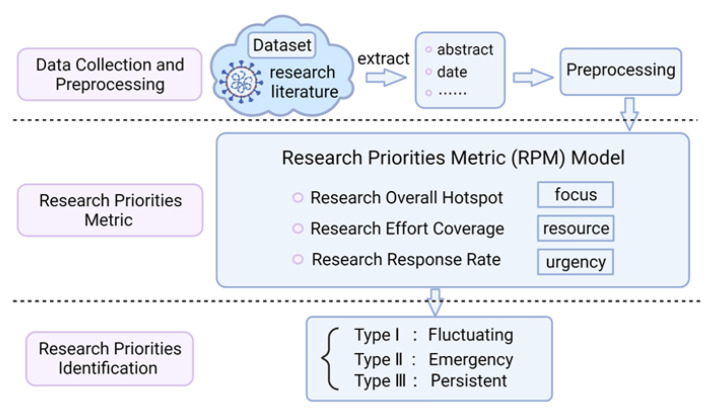
Research priorities identification framework based on scientific literature text analysis.

This paper proposed a research priorities identification framework for pandemic based on scientific literature text analysis to understand the swing of research priorities during the pandemic. A research priority metric model was established to measure the characteristics of research priority by combining the overall focus, research resources investment, and time urgency. The dynamic characteristics of research priorities in various fields during the pandemic were analyzed macroscopically, and the urgency of research priorities in each stage of the pandemic was analyzed microscopically. The specific process of this paper is shown in Figure 1.

### 2.1. Data Collection and Preprocessing

In this paper, the relevant scientific literature published during the pandemic outbreak was analyzed to identify the research priorities. Therefore, literature related to COVID-19 was selected as research data. Relevant scientific literature in appropriate databases needs to be screened according to keywords and publication date and the research data were preprocessed. 

CORD-19 is a dataset [27] that was prepared by the White House and a coalition of leading research groups in response to the COVID-19 pandemic. The dataset contains all COVID-19-related research from the following sources: (1) PubMed’s PMC open access corpus; (2) a corpus maintained by the WHO; (3) bioRxiv and medRxiv pre-prints. The information of the research in the dataset includes title, author, publication date, abstract and affiliation, and etc. This paper took the CORD-19 data set as the original data of empirical analysis. The selected paper was published from 24 January 2020 to 3 September 2020, which is the early stage of COVID-19-related research, which can intuitively reflect the changes in the research priorities after the occurrence of the pandemic. 

In order to ensure the validity of the experimental data, the data need to be preprocessed. First, the records with empty abstracts or non-English abstracts (i.e., not provided in the database) were deleted, and the final dataset contained 25,034 records. Meaningless words were deleted, such as stop words, common terms in scientific articles (e.g., “introduction” and “method”) and other words that need to be ignored. Through the Natural Language Toolkit, the abstract was lemmatized, and the lemmas with absolute frequency less than 4 and relative frequency greater than 80% were pruned. Eventually, the vector space representation R established by TF-IDF weighting was an n×m matrix, where n = 25,034 (the number of documents) and m = 19,141 (the number of lemmas).

### 2.2. Research Priorities Metric and Assessment

**Figure 2:**
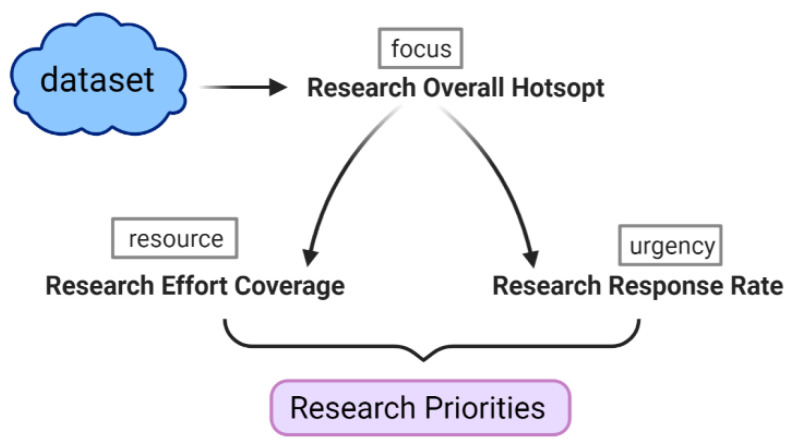
Research priorities metric model.

As shown in Figure 2, the Research Priorities Metric (RPM) model was proposed, which combines three metrics including Research Overall Hotspot, Research Effort Coverage, and Research Response Rate, to reveal the research priorities from three perspectives of overall focus, research resource investment and time urgency. Then, according to the evolution of the pandemic, the dynamic characteristic of RPM values during each stage of the pandemic were analyzed from both micro and macro levels.

#### 2.2.1. Research Overall Hotspot

Learning topics from short texts has become a critical and fundamental task for understanding the massive information, and the topic model can reveal potential topics from a large number of unstructured short texts [28]. After completing the data collection and preprocessing steps, the topic model was used to analyze the abstract of the massive pandemic-related scientific literature and discover potential topics. Non-negative matrix factorization (NMF) is a vector space decomposition method [29], which is widely used in image processing, speech processing, text mining, and other fields. NMF expects to find such two matrices W and H, so that the value of each position of the matrix obtained by the matrix product of W and H is compared with the value of the corresponding position of the original matrix R, and the error obtained is as small as possible, as shown in Equation (1). W is an n×k word matrix in which each column represents a topic. H is a topic matrix in which each row represents a word. Here, an algorithm based on alternating least square with projected gradient descent was used to solve the objective function.

(1) $$\underset{W,H}{\min}f(W,H)=\frac{1}{2}\sum_{i=1}^{n}\sum_{j=1}^{m}(R_{ij}-{\left(WH\right)}_{ij}{)}^{2}$$

In order to control the pandemic and reduce its impact, researchers carried out research in various fields related to the pandemic. In the whole research process, more attention was paid to important research topics, and a large amount of time and resources were invested in the research of these topics. These topics were called Research Overall Hotspots (ROH). By analyzing and interpreting the keywords with high weight in each topic, the topic content was summarized and refined, and gave the description label of each topic, so as to get the ROH of pandemic.

It is one of the keys to the quality of topic modeling to determine the appropriate number of topics k, so as to make the same feature words between topics less while the topic distinction higher. To get the appropriate number of topics, two metrics were combined to help select the optimal potential topic structure. First, the metric proposed by Greene et al. [30] was used to assess the stability of the potential topics identified. The average weighted Jaccard distance between the ordered word sets of a topic was used to measure the stability of the topic structure. Another metric, proposed by Arun et al. [31], described the symmetric Kullback-Liebler divergence between the singular value distribution of the matrix H and the row-l2 norm distribution of the matrix W.

#### 2.2.2. Research Effort Coverage

In order to analyze the temporal distribution of ROH during the pandemic outbreak, Research Effort Coverage (REC) metric was defined. By calculating the number and proportion of literature belonging to each hotspot in the time dimension, the ongoing research effort of each hotspot in overall research during each stage of the pandemic was described, which reflects the distribution of research resources in each field. Taking $w\in N^{+}$ days as a time-window, the literature was partitioned within the selected time period. By calculating the proportion of the literature belonging to each hotspot to the total number of published literature in each time-window, the REC can be measured as:

(2) $$REC(t,w)=\frac{\sum_{i=1}^{n}\xi (p_{i}^{d}\in w\wedge d_{i}\in D(t))}{\sum_{i=1}^{n}\xi (p_{i}^{d}\in w)}$$

where, ξ(X)=1 if X is true, otherwise, ξ(X)=0. REC(t,w) represents the REC of topic t in the time-window w, $p_{i}^{d}$ is the publication date of the *i*th literature, $d_{i}$ is the *i*th literature, D(t) is the set of literature belonging to topic t, and n is the number of literature in the whole dataset.

Then, the distribution of literature belonging to each hotspot in each time-window, that is, the distribution of ROH in time dimension was analyzed respectively, and the Mann-Kendall test [32,33] was used to determine whether the REC of each hotspot increased or decreased over time to a statistically significant degree, using the standard 95% confidence level. This method constructs the MK test statistics $Z_{MK}$ through the time series. If it is greater than 0, it means the REC has an upward trend in the time series, and if it is less than 0, it has a downward trend.

#### 2.2.3. Research Response Rate

Combined with the starting point of the COVID-19 outbreak, the publication of relevant scientific literature was further analyzed in terms of time. During the pandemic outbreak, research in various relevant fields was carried out immediately. The closer the relationship between the research field and the event, the more urgent the topic, the higher the response rate of relevant scientific literature. Then the average number of days between the starting point of the outbreak and the publication date of the literature belonging to each hotspot within each time-window was calculated as the research time distance. Research time distance can be calculated as:

(3) $$B(t,w)=\frac{\sum_{i=1}^{n}b_{i}\xi (p_{i}^{d}\in w\wedge d_{i}\in t)}{\sum_{i=1}^{n}\xi (p_{i}^{d}\in w\wedge d_{i}\in t)}$$

where, B(t,w) represents the research time distance of topic t in the time-window w, and $b_{i}$ represents the number of days between the publication date of the *i*th literature and the outbreak starting point.

At the same time, information has uncertainty. In a random event, the greater the uncertainty of the event, the greater the entropy [34]. Therefore, topic information entropy is introduced to reduce the interference of topic uncertainty. Topic information entropy can be calculated as:

(4) $$E(t,w)=-\sum_{d\in D(t,w)}p(d|t)\log p(d|t)$$

where, E(t,w) represents the topic information entropy of topic t in the time-window w, p(d|t) represents the probability that the literature d belongs to the given topic t, D(t,w) is the set of literature belonging to the topic t in the time-window w.

Then, combining research time distance and topic information entropy, the research response rate (RRR) can be calculated as:

(5) $$RRR(t,w)=\frac{1}{B(t,w)}\sum_{d\in D(t,w)}\frac{p(d|t)}{E(t,w)}$$

The RRR metric measures the publishing speed of research hotspots during the pandemic outbreak and reflects the response of various research fields. The RRR of different hotspots in the same stage were compared from the perspective of research field and found the most urgent research hotspots in each stage. At the same time, the evolution characteristics of RRR in the whole process of different hotspots were compared, and the fluctuation, emergency, and persistence of each hotspot were analyzed.

### 2.3. Research Priorities Identification

Through the above analysis, the ROH, REC, and RRR of related literature during the pandemic were obtained. In this part, combined with the changes in these three aspects, a specific analysis of the research connotation and the situation in various fields was conducted according to overall focus, research resources investment, and time urgency. The evolution characteristics of RPM values in each field in each stage of the development of the pandemic were sorted out, and the research characteristics and specific situations of each field were combined to systematically identify the research priorities of the pandemic in view of the general characteristics of urgency, significance, and uncertainty of the pandemic from macro and micro level. From the macro level, the dynamic evolution characteristics of research priorities in various related fields with the development of the pandemic were analyzed. From the micro level, the distribution of research priorities in each stage during the epidemic was analyzed, and the research areas that need to be focused on in each stage were determined. 

In the subsequent analysis, a contrastive analysis between the research priorities uncovered by the RPM model and those identified by experts was conducted, and comprehensively the effectiveness of the research priority identification method proposed in this paper was evaluated. At the same time, based on the characteristics of the pandemic, research priorities were identified after the occurrence of a universal pandemic, emergency scientific research response strategies were formed, and fine-grained suggestions were put forward for the government, hospitals, and scientific research institutions, etc., so as to accelerate research in various fields to facilitate that those affected receive optimal care, reduce social and economic losses, and prepare for the next unforeseen pandemic.

## 3. Results

### 3.1. Research Priorities Metric and Assessment

#### 3.1.1. Research Overall Hotspot

**Figure 3:**
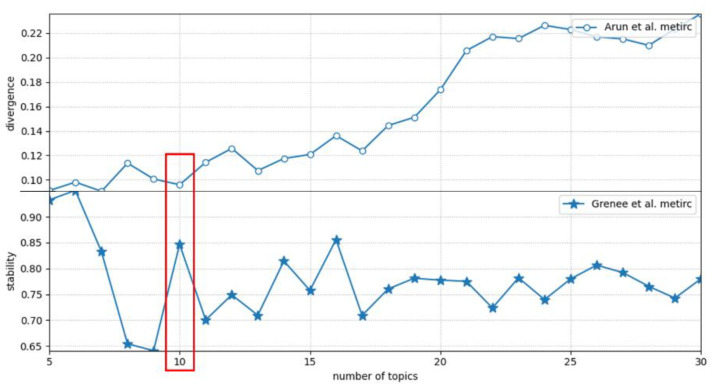
Divergence and stability for different number of topics.

We chose to use NMF for topic modeling of COVID-19-related research. When determining the number of topics k, if k is too large, the topics’ structure may be redundant and the topics will be too trivial, and if k is too small, the topics’ structure may be vague and the topics will be insignificant [24]. First, based on experience and exploration, it was estimated that the number of topics ranges from 5 to 30, and then the number of topics k was continuously adjusted with an increment of 1, and calculated the stability and symmetric KL divergence in the topic model corresponding to each value of k, and the number of topics with high stability and low divergence was selected. It can be seen from the results in Figure 3 that when the number of topics was determined as 5 and 10, the topic modeling result has low divergence and high stability. Due to a large number of papers in the dataset, when the number of topics is determined as 5, the topics’ structure is vague, which cannot well represent the hotspots of research literature during the pandemic. Finally, combining the two metrics and the discovered topic structure, the number of topics k was determined as 10, the stability value is 0.8468, the divergence value is 0.0960, and the topic structure is clear.

Through NMF, the literature was classified into 10 groups as 10 research overall hotspots for the pandemic. According to the distribution of each word in the hotspot obtained by the topic model, the words with the top 5 weights were selected as the hotspot keywords. Each group of keywords was analyzed and interpreted respectively, the content of each topic was summarized and refined, and the description labels were determined. The ROH include clinical treatment, coronavirus, nursing and health care, epidemic prevention and control, risk factors, diagnosis and testing, drugs and vaccines, social psychology, infection process and clinical characteristic. The distribution of the ROH and keywords of each hotspot is shown in Table 1.

#### 3.1.2. Research Effort Coverage

**Figure 4:**
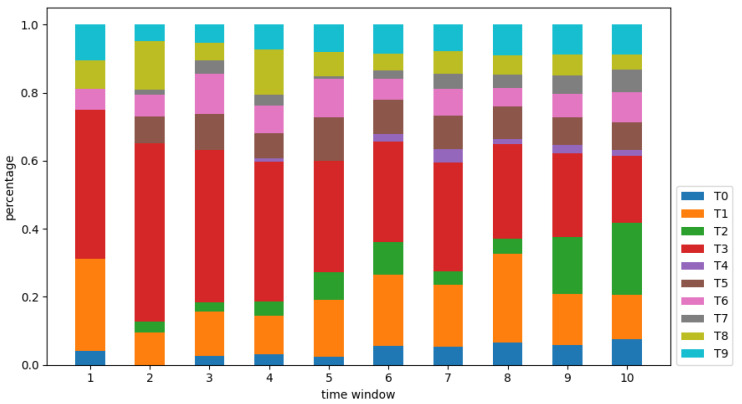
Research effort coverage.

In the light of the actual situation, 7 days were determined as the length of a time-window, the selected time period was partitioned as 10 time-windows, and the literature in each time-window was analyzed. According to Equation (2), the REC for each hotspot in 10 time-windows was calculated, and the results are shown in Figure 4. Through the Mann-Kendall trend test, it can be seen that during the whole process, T0, T2, T4, and T7 have a significant enhancement trend, in which the REC of T2 continues to increase and has become one of the most popular research topics in the later stage. It can be seen that clinical treatment, nursing and health care, risk factors and social psychology are persistent research hotspots, and continuous research resources can be invested in them. T3 and T8 decrease slightly over time, but T3 remains the most concerned at almost any time-window, indicating that the research effort of epidemic prevention and control and infection process is mainly concentrated in the early stage of the outbreak and has been quickly improved. The RECs of T1, T5, T6, and T9 maintain a stable trend, in which the research effort of T1 has always been in the top 3. Therefore, related research effort on coronavirus, diagnosis and testing, drugs and vaccines, and clinical characteristic has been proceeding in an orderly manner.

#### 3.1.3. Research Response Rate

**Figure 5:**
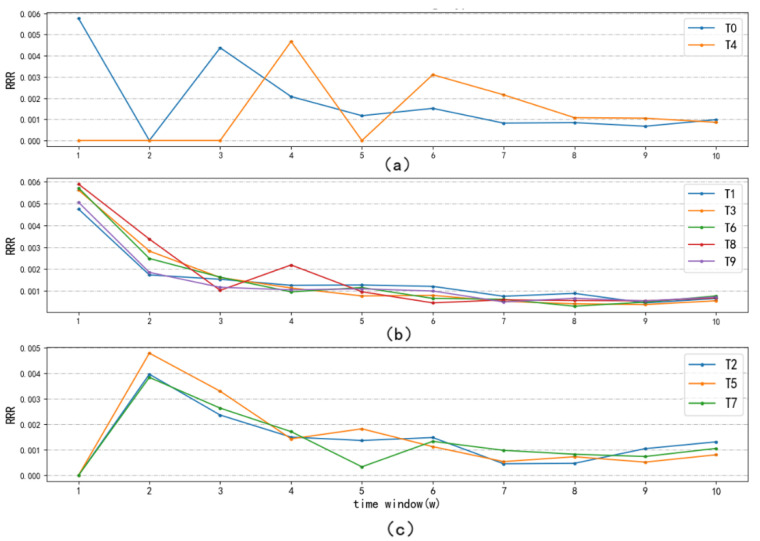
Research response rate. (**a**) The fluctuating type; (**b**) the emergency type; (**c**) the persistent type.

Taking 12 December 2019 as the starting point of the COVID-19 pandemic, the research time distance was calculated according to Equation (3). At the same time, the topic information entropy was calculated combined with the results of NMF. Then, the RRR was obtained according to Equation (5). By analyzing the RRR for 10 hotspots, three different trends were found. As shown in Figure 5a, T0 and T4 belong to the fluctuating type, their RRRs fluctuate greatly during the whole period, which may be related to the occurrence of specific events related to the pandemic. The occurrence of specific types of cases leads to corresponding research on clinical treatment and risk factors. In Figure 5b, T1, T3, T6, T8, and T9 belong to the emergency type, showing an overall downward trend. Such research hotspots are closely related to the pandemic, and need to be studied quickly and effectively to rescue more patients and curb the development of the epidemic. In Figure 5c, T2, T5, and T7 belong to the persistent type, the response speed is not fast as the emergency type, and the RRR reaches the peak in the second time-window, and then declines slowly for a long period. The research on more effective diagnosis and detection methods continues, and the research on nursing and health care and social psychology are particularly important in the middle and late stages of the pandemic. The RRRs of both emergency and persistent type are high in the early stage of the outbreak of the pandemic and the decline rate are fast, and stabilized at a low level in the later stage. These two kinds of research hotspots, as the main research contents of COVID-19, have been carried out throughout the entire process in a rapid and orderly manner.

### 3.2. Research Priorities Identification

**Figure 6:**
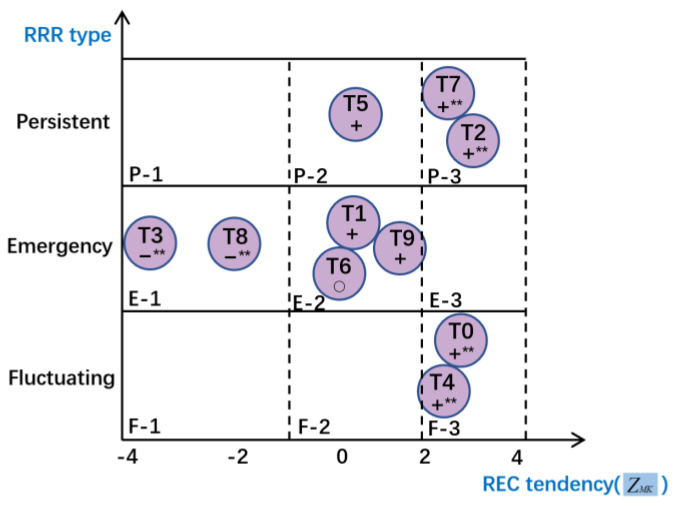
Research priorities identification for COVID-19 pandemic. “+”, “−” and “o” in the figure indicate increasing trend, decreasing trend and no change trend respectively, ** means significant at the 5% level.

Through the research priorities metric model, the characteristics of the ROH, REC, and RRR of COVID-19 relevant scientific literature during each stage of the pandemic were obtained. ROH discovers the distribution of potential research hotspots from the abstracts of all the literature. REC measures the proportion of the research effort of each hotspot in the overall research in each time period. RRR describes the dynamic evolution characteristics of the hotspot in the time dimension. Combining REC and RRR, the research priorities of the COVID-19 pandemic was identified from static and dynamic aspects. According to the RRR of research hotspots, 10 hotspots were identified as Type I: persistent, Type II: emergency and Type III: fluctuating, and through the Mann-Kendall test, the REC trends of three types were analyzed. The identification result of the research priorities of COVID-19 pandemic relevant scientific literature is shown in Figure 6.

#### 3.2.1. Fluctuating Research Hotspot

In the ten time-windows after the relevant research was published, fluctuating research hotspots include T0 and T4. Their RRR fluctuates greatly, and REC increases, but the proportion of literature belonging to this type is not high, which is located in the F-3 region in Figure 6. Such research hotspots are closely related to the medical events that occurred in the pandemic. After the emergence of new types of cases or situations, it is necessary to immediately carry out clinical treatment and risk factor-related research, and quickly obtain research results and improve the treatment plan.

When an emergency occurs, a large number of F-3 research is urgently carried out, and the research results are produced quickly and efficiently. During the outbreak, the number of confirmed patients increased rapidly in a short time, a large number of patients were waiting for treatment, and medical resources such as beds, drugs, and prevention and control materials were in short supply. Therefore, clinical treatment has become the focus of initial research. Scientific research achievements including treatment, prevention, and control were published rapidly and put into clinical practice. With the development of the pandemic, the risk factors of COVID-19, such as comorbidities and complications, attracted the attention of researchers, and relevant research was focused on. In terms of F-3 research, it is of great significance for the treatment and nursing of patients to conduct targeted research on the medical problems in the progress of emergencies, accelerate the production of results, and conduct continuous research.

#### 3.2.2. Emergency Research Hotspot

Emergency research hotspots include T1, T3, T6, T8, and T9. The overall proportion of emergency research hotspots are relatively high, and their RRRs are at the highest value in the first time-window, and then drop to a stable level and keep a certain proportion. The RECs of T1, T6, and T9 are stable and located in the E-2 region, while the RECs of T3 and T8 gradually decrease and are located in the E-1 region. Emergency research hotspots need to invest scientific research resources to carry out rapid and comprehensive research when the pandemic occurs. E-1 research is important but can be solved well in the early stage, and E-2 research needs continuous attention.

E-1 research responded quickly during the pandemic outbreak, and the research results were clear and effective. Measures to prevent and control the COVID-19 epidemic have attracted extensive attention from scholars immediately after the outbreak. Comprehensive and effective pandemic prevention research is the key to curbing the pandemic and reducing economic losses as soon as possible. In order to carry out the research on prevention and control of the pandemic, the way the virus enters cells and infects the human body has become the most urgent research topic. The strong demand for basic knowledge of epidemic prevention and treatment and early comprehensive research has led to the rapid improvement and stabilization of the research on these two hotspots, and the research attention has decreased.

E-2 research was carried out immediately during the outbreak of the pandemic, and with the progress of the pandemic, the research continued. At the beginning of the pandemic, coronavirus quickly became the priority of research to improve the overall understanding of SARS-CoV-2. Rapid and complete coronavirus research is the premise of COVID-19 pandemic medicine research, which is of guiding significance for the prevention of viral infection, clinical treatment, and vaccine development. At the same time, the analysis and report of clinical characteristics of patients with different populations and different risk diseases provide rich clinical samples for treatment, drug, and vaccine research. The research and development of drugs and vaccines is also a key part of the early pandemic research. The research on the effectiveness and safety of existing antiviral drugs and newly developed drugs has been carried out rapidly, and the continuous research on safer and more effective drugs and vaccines has played a key role in patient treatment and epidemic prevention and control.

#### 3.2.3. Persistent Research Hotspot

Persistent research hotspots include T2, T5, and T7. The response of persistent research hotspots lags behind the pandemic, and the RRR reach the peak in the second time-window, and the decline is small, these hotspots have strong persistence. The REC of T5 remained stable, and is located in the P-2 region, the RECs of T2 and T7 increase and are located in P-3 region. For a long time after the occurrence of the pandemic, persistent research hotspots are the focus of scientific research, which requires long-term human and material resources investment.

As is shown in Figure 6, the intensity of P-2 research remains stable in the middle and late stages. Since the COVID-19 pandemic is a new and unknown challenge, the response to P-2 research is slow, the process of obtaining results is longer, and attention is maintained in the middle and late stages, which continued to provide effective and accurate tools for the diagnosis of cases. 

While P-3 research is becoming more and more important and has become a significant research priority in the middle and later stages. In terms of nursing and health care, at the beginning of the epidemic, there were relatively fewer studies on people’s daily health care and post-operative care of patients in different situations, and continued to be carried out after the pandemic had gradually stabilized, which is the key measure to maintaining the prevention and control work after the pandemic is under control. Due to a large number of infected people, wide spread and high mortality rate, COVID-19-related topics can easily affect public sentiment, and social psychology has become one of the research topics of scholars. In the whole process of the pandemic, all kinds of social psychology problems became the focus of research, and the related research continue for a long time and need to be paid special attention.

As a result, through the research priorities identification framework, at the macro level, potential research hotspots were discovered from the research literature in the pandemic and divided into three types such as persistent, emergency, and fluctuating type. At the micro level, the dynamic evolution characteristics of the research hotspots in each type were identified. Therefore, the research priorities of each key stage in the development of the pandemic, especially in the early stage of the pandemic, have been systematically identified, which provide a reference for solving the key scientific and technological problems in pandemic management and prevention.

### 3.3. Research Priorities Identification Contrastive Analysis

In the COVID-19 pandemic, in order to synchronize the work and identify research gaps in cutting-edge priority areas, the World Health Organization has developed a research blueprint, including timetables and roadmaps [18]. Experts and scholars identified the key knowledge gaps and research priorities based on the actual needs and current research situation, and analyzed the state-of-the-art, knowledge gaps, ongoing research efforts, and key milestones of each research priority. In this part, a contrastive analysis between the research priorities identified by the RPM model and those identified by experts was conducted, and the research priority identification method proposed in this paper was evaluated comprehensively. 

The research priorities identified through the RPM model were compared with the research frontier areas identified in the Global Research Roadmap, which is shown in Table 2. It can be seen that the coverage of the research priorities identified through the RPM model is basically the same as that of the Global Research Roadmap, indicating that the model has practical significance in identifying the research priorities in major public health emergencies. At the same time, the clinical treatment and risk factors in the research priorities are related to candidate therapeutics R&D in the roadmap. Due to the numerous risk factors related to COVID-19 and their impact on patient treatment and care, a large number of studies have focused on risk factors, so disease-related risk factors are also one of the research priorities. The clinical characteristics and nursing and health care in the research priorities are related to the clinical characteristics and management in the roadmap. Cases of patients with different clinical characteristics are collected to study treatment methods and determine appropriate follow-up nursing and health care measures. To a certain extent, the RPM model identifies the research priorities of COVID-19 in a more fine-grained manner and analyzes the evolution of research priorities. In addition, the implementation time of the research priorities identified through the RPM model is concentrated in the early stages of COVID-19 research, ROHs belonging to emergency type and fluctuating type are prominent in February, March, and April of 2020 and ROHs belonging to persistent type are ensuing in the following months, while according to the timeline in the roadmap, the Expected Month for Completion of each research topics is relatively late. In view of the urgency and significance of the pandemic, the RPM model can be used to promote the response to major public health emergencies and the improvement of emergency scientific research capabilities.

It clearly shows that the results have guiding significance in theory and practice when applied to scientific research management and epidemic prevention and control during the pandemic. At the same time, the traditional method of determining the research direction by authoritative experts is compared with the method based on the scientific literature text analysis, so as to show the effectiveness of the research priorities identification framework in fine-grained dynamic identification of research priorities, supplement the existing scientific literature analysis methods of public health emergencies, and provide a reference for the formation of emergency scientific research response strategies.

## 4. Discussion

The three RPM values of research topics were analyzed from micro and macro levels to reveal the evolving characteristics during the pandemic. Research priorities were identified, and emergency scientific research response strategies were formed to put forward suggestions for the government, hospitals, and scientific research institutions, etc., so as to accelerate research in various fields to facilitate that those affected receive optimal care, reduce social and economic losses, and prepare for the next unforeseen pandemic.

For fluctuating research hotspots such as clinical treatment and risk factors, due to the close relationship between the research content and the development of the pandemic, the whole process of related experiments is long, so the research should be carried out in advance or be prepared to be carried out at any time as required. According to the development of the pandemic, it is of great significance to continuously explore new research contents, carry out comprehensive and effective scientific research in an orderly manner, accelerate the production of achievements, and promote the research work in the long term.

Emergency research hotspots are of great significance and urgency. At the beginning of the epidemic, people paid great attention to it. At the beginning of the epidemic, great attention has been paid to the research on virus and prevention and treatment, a large number of human and material resources have been invested in the related research, and the results have been achieved quickly and put into clinical application. We should seize all the time to save more patients’ precious lives. It is necessary to carry out a large number of relevant research at the first time, and take the research results in the shortest time as the theoretical basis for follow-up research, so as to strive for more time for patient treatment and pandemic containment, minimize social and economic losses.

Persistent research hotspots should be studied in all stages of the development of the pandemic, so as to control the development of the pandemic within the normal range, save as many people as possible during the whole process of the pandemic, and minimize social and economic losses. With the gradual deepening of the understanding of the epidemic, breakthroughs have been made in diagnosis and detection-related research, and more accurate and effective detection and diagnosis methods and technologies have been continuously pursued, which can greatly improve the progress of patient diagnosis and epidemic prevention and control. The research on nursing and health care for different groups continue to be paid attention to after the pandemic stabilizes, so as to consolidate the treatment of patients and prevent the recurrence of the pandemic. In the whole process of pandemic development, social psychology research should be carried out continuously to ensure that epidemic prevention and control and public opinion stability develop in a positive direction.

The emergency scientific research response strategies promote the rapid and orderly conduct of research, allowing decision-makers to have a comprehensive and long-term understanding of the research plan, and provide guidance on achieving goals and controlling time nodes. Combining the development characteristics of the pandemic, and considering the constraints of time and resources, the research goals of different research fields and research topics are determined. Reasonable research strategies formulate during each stage of the pandemic development, and research resources allocate scientifically to provide scientific and technological support for overcoming the huge impact of the pandemic.

## 5. Conclusions

After the occurrence of a pandemic, relevant scientific research is carried out quickly, in an orderly and efficient manner, which is the most effective way to deal with the pandemic. Combining machine learning and big data analysis, this paper proposed a research priorities identification framework for pandemic based on scientific literature text analysis. A research priority metric model was proposed to measure the characteristics of research priorities. The empirical analysis from COVID-19 scientific literature was conducted, and the research priorities during the pandemic were fine-grained and dynamically identified. Based on the results, the public health emergency scientific research response strategy was discussed to provide decision support for solving key scientific and technological issues in epidemic prevention and control. This article also has some limitations. For example, since most of the papers published in the early stages of the pandemic are in English and there are fewer papers in other languages, this article only considers papers in English. Papers published in other languages can also be considered in future research.

## Figures and Tables

**Table 1:** Research overall hotspots.

ROH Id	ROH Label	Keywords
T0	Clinical treatment	patient, hospital, group, clinical, admission
T1	Coronavirus	SARS-CoV-2, virus, rna, human, viral
T2	Nursing and health care	care, pandemic, healthcare, health, cancer
T3	Epidemic prevention and control	case, China, epidemic, 2020, number
T4	Risk factors	ci, risk, study, mortality, analysis
T5	Diagnosis and testing	ct, chest, rt-pcr, imaging, pneumonia
T6	Drugs and vaccines	drug, treatment, trial, clinical, antiviral
T7	Social psychology	health, mental, social, anxiety, psychological
T8	Infection process	ace2, angiotensin, cell, receptor, expression
T9	Clinical characteristic	respiratory, severe, acute, syndrome, coronavirus

**Table 2:** Comparative analysis of research priorities during the COVID-19 pandemic.

ROH Id	ROH Label	Research Priorities in the RPM Model	Research topics in Global Research Roadmap (GRR)	Expected Month for Com-pletion in GRR
T3	Epidemic prevention and control	Emergency (E-1)	Social sciences in the outbreak response	February-20
T8	Infection process	Emergency (E-1)		
T6	Drugs and vaccines	Emergency (E-2)	Epidemiological studies	March-20
T1	Coronavirus	Emergency (E-2)		
T0	Clinical treatment	Fluctuating (F-3)	Clinical characterization and management	
T4	Risk factors	Fluctuating (F-3)		
T9	Clinical characteristic	Emergency (E-2)	Candidate therapeutics R&D	April-20
			Candidate vaccines R&D	
T5	Diagnosis and testing	Persistent (P-2)	Virus natural history, transmission and diagnostics	June-20
T7	Social psychology	Persistent (P-3)	Animal and environmental research	July-20
T2	Nursing and health care	Persistent (P-3)	Infection prevention and control, including health care workers’ protection	August-20
			Ethics considerations for research	

## Data Availability

The data used to support the findings of this study are available from the corresponding author upon request.
